# The interrelationship between concepts about agency and students’ use of teachable-agent learning technology

**DOI:** 10.1186/s41235-019-0163-6

**Published:** 2019-04-18

**Authors:** Christopher Brett Jaeger, Alicia M. Hymel, Daniel T. Levin, Gautam Biswas, Natalie Paul, John Kinnebrew

**Affiliations:** 1 Department of Psychology and Human Development, Vanderbilt University, 230 Appleton Place, Nashville, TN 37203-5701 USA; 2 Department of Electrical Engineering and Computer Science, Vanderbilt University, Box 1824, Station B,, Nashville, TN 37325 USA; 3 mabl, 141 Tremont Street, Boston, MA 02111 USA

**Keywords:** Agency, Theory of mind, Learning technology, Metacognition

## Abstract

To successfully interact with software agents, people must call upon basic concepts about goals and intentionality and strategically deploy these concepts in a range of circumstances where specific entailments may or may not apply. We hypothesize that people who can effectively deploy agency concepts in new situations will be more effective in interactions with software agents. Further, we posit that interacting with a software agent can itself refine a person’s deployment of agency concepts. We investigated this reciprocal relationship in one particularly important context: the classroom. In three experiments we examined connections between middle school students’ concepts about agency and their success learning from a teachable-agent-based computer system called “Betty’s Brain”. We found that the students who made more intentional behavioral predictions about humans learned more effectively from the system. We also found that students who used the Betty’s Brain system distinguished human behavior from machine behavior more strongly than students who did not. We conclude that the ability to effectively deploy agency concepts both supports, and is refined by, interactions with software agents.

**Electronic supplementary material:**

The online version of this article (10.1186/s41235-019-0163-6) contains supplementary material, which is available to authorized users.

## Significance

In recent years, we have seen a steady stream of new, increasingly intelligent technologies intended to improve our lives in various ways. One important forum for these technologies is the classroom, where teachable agent software is used to help students learn. A teachable agent is a graphical character in a computer environment that can be taught concepts by students and then, using artificial intelligence, answer questions, complete quizzes, and provide explanations based on what the student has taught it. The idea is that explaining material to teachable agents might provide students with educational benefits similar to those obtained by explaining material to other students.

But teachable agents are not other students. Interacting with these agents can be challenging, because they behave in some respects like humans and in other respects like machines. We found that students who demonstrated a stronger understanding of human intentionality on a behavioral prediction measure learned more effectively from teachable agent software. We also found that the process of interacting with teachable agents can influence how students deploy agency concepts. Together, these findings suggest an important reciprocal relationship between students' use of software agents and students' understanding of them.

## Introduction

The rapid technological development of the past two decades has spawned a variety of software agents that can perceive and act with some degree of autonomy (Rudowsky, 2004; Russel & Norvig, 2010; Woolridge & Jennings, 1995). When people interact with these software agents, they may call upon many of the cognitive skills that underlie human-to-human interaction (e.g., Kuchenbrandt, Eyssel, Bobinger, & Neufeld, 2013; Malle, 2015). People may, for example, have to interpret a software agent’s request, reason about its goals, or make predictions about its behavior. But the unique properties of software agents can make these tasks challenging. Software agents, by design, behave in some respects like humans but in other respects like machines, and different software agents may reflect different aspects of human thought. As a result, interacting with software agents requires people both to call upon concepts of how human and mechanical agents operate and to deploy these concepts effectively given the pragmatics of the interaction.

For example, when one encounters a software agent during a service call, a successful interaction requires more than a simple decision of whether to treat the agent as a person or a machine. It also requires explicit consideration of particular ways in which the agent is likely to be person-like. For instance, the service-call agent may have some forms of knowledge and may be able to respond to emotions, but it is unlikely to know much about topics irrelevant to the typical service call or to have non-auditory sensory functions.

Further, your interaction with the automated system may elicit responses that are incompatible with how you thought the system was operating. These responses will help you calibrate how you conceptualize this particular automated system, and they may also help you refine your deployment of agency concepts in future interactions with other systems (Epley, Waytz, & Cacioppo, 2007; Gopnik & Wellman, 1994; Levin, Saylor, Adams, & Biswas, 2013; Levin, Saylor, & Lynn, 2012). This form of learning about agents has rarely been explored empirically, but it may be quite important, especially given recent arguments that it could induce a fundamental change in our understanding of the ontological distinction between living and nonliving things (Kahn et al., 2012).

In this paper, we examine the reciprocal relationship between agency concepts and agent interactions in one particularly important context: the classroom. We report three experiments in which middle school students used an established teachable-agent-based computer learning environment called Betty’s Brain (Blair, Schwartz, Biswas, & Leelawong, 2007; Leelawong & Biswas, 2008) for lessons on scientific topics. We find that students with stronger pragmatic understanding of human agency—that is, students who make more intentional predictions about human behavior on a behavioral prediction measure—learn more effectively from the teachable agent system. We also find that the process of interacting with the system sharpened students’ distinctions between human behavior and mechanical behavior.

### Conceptualizing agents

When considering how people conceptualize software agents, it is helpful to start with a definition of “software agents”. Although researchers have relied upon a range of definitions, one relatively uncontroversial definition is that software agents are programmed entities that include some form of autonomy, ability to learn, and ability to interact socially with human users (see Nwana, 1996 for review). As this definition suggests, when users need to understand software agents, they likely draw upon their understandings of human thinking.

A variety of findings suggest that, when an unfamiliar entity exhibits minimal cues of agency (e.g., an entity has “eyes”, appears to make goal-directed movements, or behaves unpredictably), people are quick to anthropomorphize it, using their knowledge about human thought and behavior as a framework for understanding and drawing inferences about the entity’s internal operations (Barrett & Lanman, 2008; Epley et al., 2007; Gray, Gray, & Wegner, 2007; Heider & Simmel, 1944; Jipson & Gelman, 2007; Kahn et al., 2012; Levin, Saylor, Adams, & Biswas, 2013; Levin et al., 2012; Martini, Gonzalez, & Wiese, 2016; Melson et al., 2009). For example, when asked to describe shapes moving around a screen in a pre-determined pattern, people tend to do so in human, goal-oriented terms, saying things like “the big triangle was chasing the little one” or “the big triangle is aggressive” (Heider & Simmel, 1944). On one view, this can be understood as extending “theory of mind” to perceived agents, imputing beliefs, desires, and goals that can explain and support predictions about their behavior (Baron-Cohen, Leslie, & Frith, 1985; Gopnik & Wellman, 1992, 1994; Wimmer & Perner, 1983).

A key concept underlying theory of mind is the distinction between intentional and nonintentional representations. Intentional representations are characteristic of human thought and are closely linked to their referents. One referent cannot be freely substituted for another, as the representation–referent link is embedded in a rich set of contextual knowledge and perceptual experiences (Dennett, 1991). Non-intentional representations, on the other hand, are more characteristic of computers. These representations are less closely linked to their referents, serving as symbolic placeholders that the system acts upon with little importance placed on their semantic content (Searle, 1986). One way of summarizing this contrast is to suggest that intentional representations reflect truly situated semantic knowledge about the world while non-intentional representations are more like pointers to a representing system that does not really “know” the true meaning of the representations. In this paper, we refer to the ability or tendency of an entity to use, or behave as though it is using, intentional representations as “agency” (Schlosser, 2015). Speaking generally, the use of intentional representations enables agents to engage in the types of coherent, goal-directed behavior characteristic of humans, while the use of non-intentional representations does not.

Although the distinction between intentional and nonintentional representations is abstract, it is possible to understand it more concretely by considering how children begin to generate different expectations for humans and inanimate objects over their first few years of life (Kuhlmeier, Bloom, & Wynn, 2004; Spelke, Phillips, & Woodward, 1995; Woodward, 1998). For example, Woodward (1998) repeatedly showed nine-month-old infants either a human hand or an inanimate reaching device (e.g., a stick) moving toward one of a pair of objects (a teddy bear or a ball). Then, on the critical trial, the locations of the two objects were switched, and the hand or inanimate stick either moved toward the same object in its new location or toward the other object in the same location. Woodward hypothesized that when the hand moves toward the previously reached-for object in the new location, the action is explainable based on a goal that is supported by an intentional representation of the object (the person wants *that object*). Alternatively, the hand that moves toward a different object in the previously reached-for location is behaving consistently with non-intentional representations: rather than acting upon a particular object, this agent is repeatedly acting on a location, meaning that the goal object can be freely substituted across trials without consequence. Woodward found that infants viewing the critical trial looked longer (indicating surprise) when the hand moved to the new object at the old location, suggesting that the infants interpreted the reach by the hand as a goal-driven intentional action. Importantly, the same action by an inanimate stick produced no such effect, implying that the infants were limiting the inference of goal-directed intentional action to the human agent.

While much research has demonstrated that children develop the basic concepts of goal-directedness and theory of mind at young ages, this does not mean that these concepts are fully elaborated or that they are consistently applied to new situations (Birch & Bloom, 2007; Christensen & Michael, 2016; Keysar, Lin, & Barr, 2003). Indeed, there are reliably measurable individual differences both in older childrens’ (Baron-Cohen, O’Riordan, Stone, Jones, & Plaisted, 1999) and adults’ (Baron-Cohen & Wheelwright, 2004) theory of mind, an observation that led Apperly (2012) to emphasize the importance of “the varying capacity to deploy [theory of mind] concepts in a timely and contextually appropriate manner” (p. 385).

Similarly, people likely vary in their capacity to apply these concepts in support of interactions with *artificial* agents such as software agents. We propose two factors that may be particularly relevant to this capacity. First, on the assumption that people use their understanding of human cognition as a base for understanding artificial agents (Epley et al., 2007), they must know enough about the set of skills that comprise human cognition to explicitly judge which of these skills a given software agent may possess. This is important because software agents vary considerably—they may simulate intentions but lack emotion, they may simulate knowledge but lack any capability of making decisions “on their own”, and they may be able to “think” in some ways but be unable to sense information in their surroundings. Having a good understanding of these skills and the dividing lines between them can prevent users from over- or under-generalizing when considering evidence about a software agent’s capabilities. Second, people must have some sense of how the pragmatics of different situations will call upon these various skills. For example, in a situation where an intelligent animated software agent assists with a word processing program, the user would benefit from understanding that the software agent’s role will require it to have knowledge about word processing and to make decisions about whether to interrupt the user with hints, but will not require the agent to possess emotions or the ability to see.

People who more easily recognize the purposes that software agents serve and the subset of human-like skills most relevant to those purposes will likely interact with software agents more effectively for a number of reasons. First, people with these abilities may be better able to “get inside the head” of a software agent, and therefore reap benefits analogous to those afforded by theory of mind in human-to-human social interactions. Second, if people cannot judge the subset of skills that a software agent is likely to exhibit in a given setting, they may become frustrated with agents that lack expected skills, or, conversely, with agents that do more than expected (de Graaf, Ben Allouch, & van Dijk, 2016; Scheeff, Pinto, Rahardja, Snibbe, & Tow, 2002). Further, even in the absence of a negative emotional response, poor pragmatic understanding of agents may cause cognitive inefficiencies, either as a result of engaging in capacity-absorbing social responses that do not facilitate problem solving (Herberg, Levin, & Saylor, 2012), or by engaging in cognitive elaborations on agents that interfere with more basic information processing (Baker, Hymel, & Levin, 2016).

### Measuring pragmatic understanding of agency

In previous work, we constructed and validated a measure of how readily adults deploy knowledge about different types of agents to novel situations. This measure asks participants to predict the behavior of multiple types of agents (e.g., a human, a computer, and a robot) in a series of scenarios (e.g., Levin, Killingsworth, Saylor, Gordon, & Kawamura, 2013). The scenarios were designed so that participants’ behavioral predictions would differ depending on whether the agent exhibited intentional, goal-directed behavior or non-intentional, mechanical behavior. For example, one of the scenarios—the “object-goal” scenario—closely followed Woodward’s (1998) experiment, asking participants to imagine that a particular agent had repeatedly selected one of two objects and then asking which object the agent would select when the locations of the two objects were switched. If participants believe the agent to be acting in an intentional and goal-directed manner, they should predict that the agent will maintain the same goal (choose the same object), but if participants believe the agent to be acting in a rote or non-intentional manner, they should predict the agent would maintain its movement pattern without regard to goal state by reaching to the new object at the old location. Other scenarios focused on categorization, with participants predicting whether agents would classify objects using taxonomic categories (for example, “office supplies”), or more surface-level, feature-based categories (for example, “rectilinear objects”; Bloom, 1997; Deak & Bauer, 1996).

On average, adult participants made more “intentional” behavioral predictions for the human and more “non-intentional” behavioral predictions for mechanical agents, providing evidence of construct validity (Levin, Saylor, et al., 2013). Additional work has demonstrated the robustness of the pattern of predictions (Levin, Harriott, Paul, Zhang, & Adams, 2013) and demonstrated that neither perceived limits in current technology nor perceived intelligence of the agents fully accounts for the differences in predictions, as the same pattern occurs when participants consider agents from the distant future (Levin, Killingsworth, & Saylor, 2008).

Importantly, however, this pattern of predictions is not obvious or universal, even among adults. While some participants consistently made “intentional” predictions for humans and “non-intentional” predictions for mechanical agents, others did not. This variability demonstrates that, although basic concepts of goal-directed behavior are typically in place—and limited to humans—at a young age (e.g., Woodward, 1998), elaboration and effective deployment of these concepts varies across development and into adulthood (Apperly, 2012; Keysar et al., 2003).

A key feature of the behavioral prediction measure is that “correct” responses require explicit recognition that the situation tests a specific agency concept. For example, consider the object-goal scenario based on the Woodward (1998) study. In the case of a human, the nominally correct prediction is that the person will maintain the same goal and reach to the old object, now in a new location. At some level, this is a simple prediction that relies on basic concepts of goal-directedness that even infants understand (Woodward, 1998). But the basic concept of goal-directedness does not totally determine the agent’s actions in this situation because a person could, for some reason, have the goal of reaching to a given location (for example, to change the weight distribution on the table where the objects rest). A correct response requires recognizing not only that such a goal would be atypical, but also that the pragmatic intent of the question is to assess typical goals. We observe that this need to coordinate basic knowledge about goal-directedness with the pragmatics of a specific situation is similar to the demands that teachable agents place upon learners in educational contexts. Specifically, in the case of a teachable agent, it is likely helpful for learners to understand how the pedagogical setting that the agent inhabits constitutes a pragmatic constraint that determines how the agent’s mental processes will operate. For example, it is useful for students to effectively merge their understanding that the Betty system is meant to teach causal biological relationships with their understanding that Betty can be said to have the goal of learning biology, while she does not have goals related to cultivating personal relationships or getting lunch.

Finally, we note that participants’ behavioral predictions can be modified by experience with agents (Levin et al., 2008; Levin, Harriott, et al., 2013). This is consistent with more general evidence suggesting that experience may increase or decrease attributions of agency to machines (Nass & Moon, 2000; Somanader, Saylor, & Levin, 2011; for review, see Epley et al., 2007; Jaeger & Levin, 2016). This is also an important component of pragmatic understanding of agency: people can re-calibrate the concepts they apply to a particular agent based on cues from the agent and the environment. The studies we report in this article investigate how experience with software agents affects understanding of agency as well as the reverse relationship.

### Teachable agents and pragmatic understanding of agency

The present studies focus on the role that pragmatic understanding of agency plays in the middle school classroom, where several well-known technology-based learning systems seek to help students learn material by having them teach it to “teachable agents”. A teachable agent is a graphical character in a computer environment that can be taught concepts by students. Using artificial intelligence, the teachable agent answers questions, completes quizzes, and provides explanations based entirely on what the student has taught it (for review, see Chase, Chin, Oppezzo, & Schwartz, 2009). The teachable agent is often viewed as an extension of the learning-by-teaching paradigm in which students not only learn more effectively by providing explanations to other students (Webb, 1983), but also learn more effectively by merely preparing to explain material to other students (Bargh & Schul, 1980; Bransford, Brophy, & Williams, 2000).

Schwartz et al. (2009) argue that learning by teaching is effective because it invokes metacognitive monitoring of both the student’s own knowledge and their partner’s (i.e., the teachable agent’s) knowledge. Betty’s Brain, an extensively studied teachable agent system, is thought to be an effective teaching tool because it invokes this type of metacognitive monitoring, among other reasons (for review, see Blair et al., 2007).^1^

The use of teachable agent software, however, may present unique challenges for some students. Students with weaker pragmatic understanding of agency may be at a disadvantage because they are less able to optimally deploy agency concepts that facilitate learning. Further, the cognitive effort involved in deploying the appropriate concepts at the appropriate times, along with the effort of monitoring cues from the software to determine which concepts are most helpful in what circumstances, may disproportionately tax the cognitive resources of these students. This would leave fewer resources available for the metacognitive monitoring critical to the learning-by-teaching paradigm—monitoring which is itself resource-intensive (Schwartz et al., 2009).

For these reasons, we hypothesize that pragmatic understanding of agency facilitates learning from teachable agent systems. There are, however, several possibilities as to *which* understandings of agency (understandings about a human, a computer, or a particular teachable agent) are most relevant to learning. Students’ understandings of *human agents* are the broadest and most commonly used. Indeed, understanding of human intentionality underlies much of everyday thought and provides a foundation for theory of mind. Students generally have far more knowledge about how people operate than how computers or teachable agents operate. The ability to draw on this broad knowledge of human intentionality should help students interact with software agents like Betty, who are in many ways designed to imitate humans. It is also possible, however, that students’ understandings of *computers* facilitate learning: teachable agents are, ultimately, symbols in computer systems. It may be that understanding the non-intentional representations used by computer systems helps students navigate some of the teachable agents’ limitations. Another interesting possibility is that students’ understandings of the *particular* teachable agent (e.g. Betty in Betty’s Brain) are most relevant to learning—at least if the students’ experience with the teachable agent system allows them to build a robust understanding of the particular agent. Further, it seems plausible that learning could be facilitated by either an intentional understanding of Betty (enabling students to better “play along” with the idea that Betty is human in the context of the software) or a non-intentional understanding of Betty (by enabling students to better recognize and cope with some of Betty’s limitations in the learning environment).

We also hypothesize that using teachable agent software will improve students’ pragmatic understandings of agency (Levin, Saylor, et al., 2013). Specifically, we expect that time spent dealing with software agents—and grappling with the attendant difficulties in deploying the appropriate agency concepts—will help refine students’ agency concepts, and their deployment of those concepts in other contexts. Again, there are a number of ways this learning could manifest itself. One possibility is that it could increase intentional attributions to Betty as students’ interactions with her increase their attributions of her agency. This would be consistent with research from Nass and Moon (2000) documenting automatic social responses caused by interactions with computers. However, it is also possible that students will learn about some of the differences between people and artificial agents as they come to know Betty’s limitations. In such a case, one might expect a decrease in attributions of agency to Betty, and possibly even an increase in attributions of agency to people as the interaction clarifies for students the salience of goals in everyday behavior.

## Experiment 1

In Experiment 1, students covered class material on climate change and food webs either through the Betty’s Brain teachable agent system (experimental condition) or traditional classroom teaching methods (control condition). We measured students’ learning through the use of pre- and posttests of the covered content.^2^ After submitting the content posttest, students completed our behavioral prediction measure, making predictions about each of a human, a computer, and Betty. Finally, students completed a property attribution questionnaire, which assessed the extent to which students attributed human-like abilities (e.g., the ability to think, to see, or to feel) to a computer (all students) and to Betty (students in experimental condition). This questionnaire was added to test whether any observed relationships between the behavioral prediction measure and learning would remain when controlling for broader attributions of intelligence and knowledge to computers and/or the Betty system. We also wanted to assess any differences in attributions of intelligence and knowledge between Betty and computers in general.

### Method

#### Participants

Participants were recruited from five classrooms in a Nashville, Tennessee public middle school. A total of 108 seventh graders (57 experimental and 51 control) were enrolled in the study, and 74 students (69%) completed all measures. Because measures were given on different days, specific analyses may include different numbers of participants. Age and sex were not collected from the participants. Informed consent was obtained from all students and at least one legal guardian of each student.

#### Materials

##### Betty’s Brain teachable agent system

**Fig. 1:**
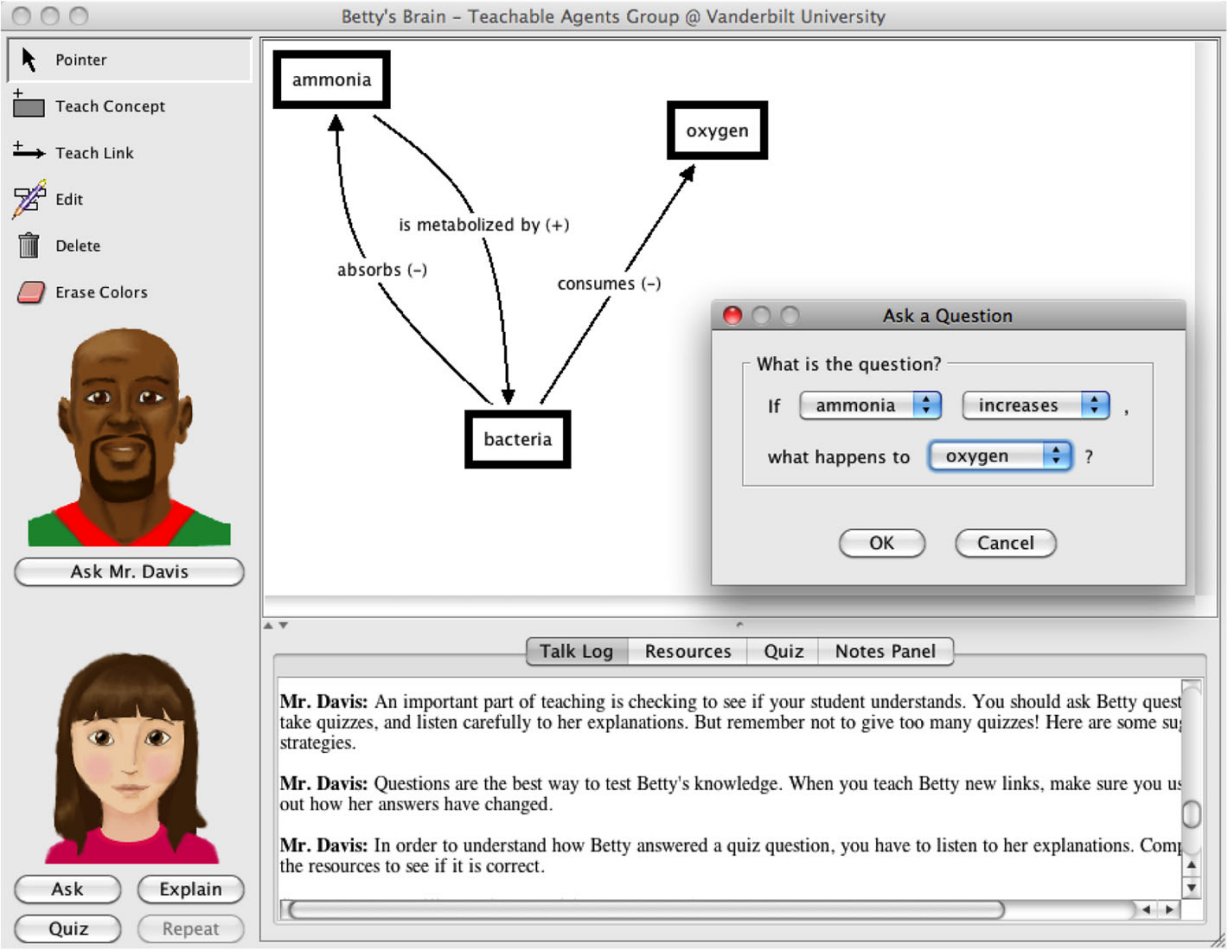
The Betty’s Brain user interface, featuring the Betty and Mr. Davis agents, student-created causal concept map, pop-up window used to ask Betty questions, and sample conversation with Mr. Davis

Betty’s Brain is a software-based learning environment in which students create causal concept maps to teach Betty, an interactive teachable agent. The software was designed to promote and reinforce metacognitive techniques, such as knowledge state monitoring, as students must ensure that Betty “understands” the material sufficiently for her to perform well on quizzes. Students use the Betty’s Brain program by reading provided texts and identifying the causal relationships among concepts described in those texts. Students put together concepts and their causal relationships by using a visual interface to create a concept map (Fig. 1). In the concept map, students create nodes that represent a concept and draw links between concepts to specify relationships. Students are able to ask Betty questions about the relationship between concepts (e.g., if A increases, what happens to B?), and Betty answers based on the current concept map. Students are also able to direct Betty to take “quizzes”—sets of questions made up by a mentor agent named Mr. Davis. Mr. Davis grades the quizzes and lets Betty and the student know which answers are right and wrong. This exercise of iteratively constructing knowledge, checking its correctness, and then revising the knowledge has been shown to improve learning (Biswas, Leelawong, Schwartz, & Vye, 2005; Leelawong & Biswas, 2008; Leelawong, Davis, et al., 2002; Segedy, Kinnebrew, & Biswas, 2013).

Importantly, Betty is designed to simulate, in some ways, a human student. Betty is represented by an animated face, and she interacts with students in a variety of ways that suggest she is a self-motivated agent, with her own beliefs, desires, and goals. Betty encourages students to read the resources and learn new information so they can teach it to her. She initiates conversations with students by restating recently taught knowledge and describing how that knowledge affects her broader understanding of the relevant material (i.e., the causal chains in the students’ concept map). Betty monitors her learning and spontaneously expresses concern (whether correct or incorrect) that what she is learning does not appear to make sense (Blair et al., 2007). Betty also requests that students ask her questions to ensure she understands and can apply the new causal relations they have taught her. Students can ask Betty to explain how she derives her answers, and she responds using speech, animation, and text. Betty expresses a desire to improve her scores on quizzes and disappointment if this goal is not met.

Of course, while Betty’s behavior appears in some ways human-like, it is in other ways mechanical. For example, while Betty has a face, her facial expression is not variable. Betty’s mood and motivation level remain constant throughout the learning session. And, of course, Betty’s knowledge is constrained by what students input in their concept maps. When students ask Betty questions, her answers are always logically drawn from the student’s concept map (she uses a qualitative reasoning algorithm described in Leelawong & Biswas, 2008).

##### Behavioral Prediction Questionnaire

After completing lessons on the Betty’s Brain system, students completed a pencil-and-paper behavioral prediction questionnaire (adapted from the behavioral prediction measure described above) to assess their pragmatic understanding of agency. The first page of the questionnaire contained pictures and a short description of each of three agents: a human, a computer, and Betty. Students then responded to three prediction scenarios for each of the three agents, a total of nine scenarios. Examples of the behavioral prediction scenarios are included in Additional file 1 (one sample scenario is included for each of the three agents).

We summed the total number of intentional predictions each student made for each agent, producing, for each student, three outcome scores ranging from 0 to 3. For example, a student who made all intentional behavioral predictions for a person and all non-intentional behavioral predictions for a computer and for Betty would have a score of 3 for person behavioral predictions, a score of 0 for computer behavioral predictions, and a score of 0 for Betty behavioral predictions.

##### Property Attribution Questionnaire

Students also completed a property attribution questionnaire, which was created for this study but drew upon similar questionnaires used by Baker et al. (2016) and Epley, Akalis, Ways, and Cacioppo (2008). This questionnaire assessed students’ beliefs about the capabilities of a computer—whether it can see, think, remember, count, feel (emotionally), know things, have intelligence, and understand a person’s desires. Students in the experimental condition also responded to the same set of questions about Betty. Students responded to all but the “know”, “intelligence”, and “desire” items on a four-point Likert scale, ranging from “definitely cannot” to “definitely can”. For the “know” and “intelligence” items, the response options compared the agent’s capabilities to a human’s using a five-point Likert scale ranging from “less than a human” to “more than a human.” The “desire” item used a three-point Likert scale ranging from 1, which indicated a high level of understanding of human desires, to 3, which indicated a low level. For this question, students were asked to consider whether a computer (or Betty) would be “able to understand what you were thinking about. For example, your friend might understand that you are looking forward to your birthday, or that you would like to get a good grade on your homework. Do you think that a computer could understand things like this about you?”

#### Procedure

Students were assigned by classroom into either the experimental or control condition. Students covered the same course material in both conditions: one unit on arctic climate change and one unit on aquatic food webs. Students in both conditions first took a content pretest to establish their baseline knowledge of arctic climate change. The content pretest included both a multiple-choice component and a short-answer component. The multiple-choice component consisted of 14 multiple choice questions of varying degrees of difficulty, and students could earn between 0 and 34 points based on their responses.^3^ The two short-answer questions asked students to explain, step-by-step, the relationship between causes and effects of climate change, and students could earn up to 11 points by identifying links in the causal chains. Samples of multiple-choice and short-answer questions from the unit on arctic climate change are included in Additional file 1. After a brief introduction to arctic climate change, students in the experimental condition underwent one class period of training in the Betty’s Brain program, while students in the control condition continued with normal lessons. Experimental students then spent four full class periods constructing their concept maps and teaching Betty, while control group students spent the same amount of time doing traditional textbook-based exercises taught by their regular classroom teachers using their preferred approach. After completing these lessons, students in both conditions took a content posttest identical to the pretest. Both groups then repeated the series of activities for the aquatic food web lessons.

After completing the content posttest for the second unit, students were given the behavioral prediction questionnaire asking them to make predictions about a human, a computer, and Betty. Control participants, who had no previous exposure to Betty, were given a brief description of Betty before completing the questionnaire. Specifically, these students were told that Betty is an animated character and that she is part of a computer program that helps students learn by teaching things to her.

Finally, students responded to the property attribution questionnaire. Those in the control condition rated a computer and those in the experimental condition rated both a computer and Betty.

### Results

#### Behavioral predictions

To examine how participants’ behavioral predictions varied across agents and conditions, we conducted a 2 × 3 mixed ANOVA. The ANOVA included condition (control vs. experimental) as a between-subjects factor, agent type (human vs. computer vs. Betty) as a within-subjects factor, and intentional behavioral predictions as the dependent variable. We found no main effect of condition (F(1,71) = 0.046, *p* = 0.83) and a significant main effect of agent type (F(2,142) = 11.521, *p* < 0.001). With respect to agent type, post hoc comparisons revealed that students made more intentional behavioral predictions for the human agent (*M =* 63%, 1.88 of 3) than for Betty (*M* = 41%, 1.22 of 3; Bonferroni-corrected *p* < 0.001) or for the computer (*M* = 43%, 1.29 of 3; Bonferroni-corrected *p* = 0.002).

We were also interested in whether students in the experimental condition (those who interacted with Betty) showed greater pragmatic understanding of agency—that is, a greater tendency to distinguish humans from machines on the behavioral prediction measure—than students in the control condition. Our ANOVA revealed no significant interaction between condition and agent (F(2, 142) = 0.85, *p* = 0.36). However, we observed that, descriptively, participants in the experimental condition made more intentional predictions for the person (65% vs. 59%) and fewer intentional predictions for both Betty (39% vs. 42%) and the computer (40% vs. 47%) than participants in the control condition. Table 1 provides a summary of participants’ behavioral predictions split by condition and agent.

**Table 1:** Mean percentage of intentional behavioral predictions (out of 3) made by participants for each agent (a human, a computer, and Betty) in each condition (control vs. Betty), for all three experiments

	Human	Computer	Betty
Experiment 1			
Control (*N* = 31)	59%	47%	42%
Betty (*N* = 42)	65%	40%	39%
Experiment 2			
Control (*N* = 39)	52%	49%	52%
Betty (*N* = 97)	67%	47%	42%
Experiment 3			
Betty (*N* = 75)	72%	43%	50%

#### Behavioral predictions and learning

Students’ performance on the content pre- and posttest is summarized in Table 2. Students in both conditions generally performed better on the posttest than the pretest, reflecting learning. Specifically, paired *t* tests revealed that students in the Betty condition improved from pretest to posttest on multiple choice questions (t(51) = 4.21, *p* < 0.001) and short answer questions (t(51) = 2.25, *p* = 0.03). Students in the control condition also improved from pretest to posttest on the multiple choice questions (t(47) = 2.72, *p* = 0.009), but did not improve on the short answer component (t(47) = 1.57, *p* = 0.12).

**Table 2:** Mean scores on content pretests and content posttests (multiple choice components (*MC*), short answer components (*SA*), and overall content scores (*OC*)) by group (control vs. experimental (Betty)) for Experiments 1–3

	MC Pre	MC Post	SA Pre	SA Post	OC Pre	OC Post
Experiment 1						
Control (*N* = 51)	15.30 (5.95)	16.81 (7.54)	1.46 (1.45)	1.72 (1.62)	0.04 (.81)	− 0.02 (.86)
Betty (*N* = 57)	15.11 (5.71)	17.60 (6.49)	1.20 (1.54)	1.51 (1.51)	− 0.05 (.78)	0.02 (.79)
Experiment 2						
Control (*N* = 39)	5.51 (2.20)	6.63 (2.47)	2.37 (1.55)	2.08 (1.86)	− 0.14 (.67)	− 0.27 (.77)
Betty (*N* = 97)	6.77 (2.51)	7.82 (2.77)	2.92 (2.07)	3.07 (2.32)	0.02 (.94)	0.07 (.91)
Experiment 3						
Betty (*N* = 75)	5.28 (2.23)	6.68 (2.21)	3.16 (2.18)	4.62 (2.87)	0.00 (.84)	0.00 (.90)

Standard deviations appear in parentheses after means. The overall content (OC) scores reflect averages of students’ standardized scores on the multiple choice and short answer components

We tested whether students’ behavioral predictions were related to their learning by running three regressions. Each regression predicted students’ posttest content scores using three predictor variables: content pretest scores, condition (control vs. experimental), and intentional behavioral prediction for one of the three agents (human, computer, or Betty). Pretest and posttest content scores reflected averages of students’ standardized scores on the multiple choice and short answer components.

As reported in Table 3, we found that behavioral predictions for a person (but not for Betty or for a computer) significantly predicted learning. That is, students who made more intentional behavioral predictions for a person also performed better on their content posttests (controlling for their content pretests). To evaluate whether the relationship between person behavioral predictions and learning differed across our experimental conditions, we re-ran the regression with a prediction–condition interaction term added to the regression model. The interaction term was not statistically significant (β = 0.109, *p* = 0.308). However, analyses run separately on the control and experimental (Betty’s Brain) conditions tentatively suggest that person behavioral predictions may be more predictive of learning in the experimental condition. As shown in Table 3, person behavioral predictions significantly predicted learning in the experimental condition but not in the control condition.

**Table 3:** Regressions predicting content posttest scores using content pretest scores, condition, and intentional behavioral predictions for particular agents in Experiment 1

	Beta	t	p
Person behavioral predictions			
Overall R^2^ = 0.678, F(3,69) = 48.410, *p* < 0.001			
Person behav. pred.	0.218	3.097	0.003
Pretest	0.749	10.709	<0.001
Condition	0.042	0.606	0.547
Betty condition only R^2^ = 0.748, F(2,38) = 56.292, *p* < 0.001			
Person behav. pred.	0.267	3.245	0.002
Pretest	0.788	9.579	<0.001
Control condition only R^2^ = 0.585, F(2,29) = 20.475, *p* < 0.001			
Person behav. pred.	0.150	1.186	0.245
Pretest	0.703	5.551	<0.001
Betty behavioral predictions			
Overall R^2^ = 0.637, F(3,69) = 40.295, *p* < 0.001			
Betty behav. pred.	0.059	0.814	0.419
Pretest	0.791	10.880	<0.001
Condition	0.069	0.946	0.347
Computer behavioral predictions			
Overall R^2^ = 0.636, F(3,69) = 40.160, *p* < 0.001			
Computer behav. pred.	0.053	0.716	0.476
Pretest	0.802	10.935	<0.001
Condition	0.072	0.987	0.327

The “Betty condition only” and “Control condition only” follow-up regressions include only participants in the named condition

We ran two additional regressions to probe whether the predictiveness of person behavioral predictions was specific to either the multiple choice or short answer components of the posttest. These regressions used students’ standardized sub-scores on the separate components of the posttest as outcome variables (and controlled for the corresponding pretest sub-score). Intentional behavioral predictions for a person were a significant predictor of learning on both the multiple choice (β = 0.229, *p* = 0.004) and short answer (β = 0.207, *p* = 0.027) components of the test.

#### Property attribution questionnaire

**Fig. 2:**
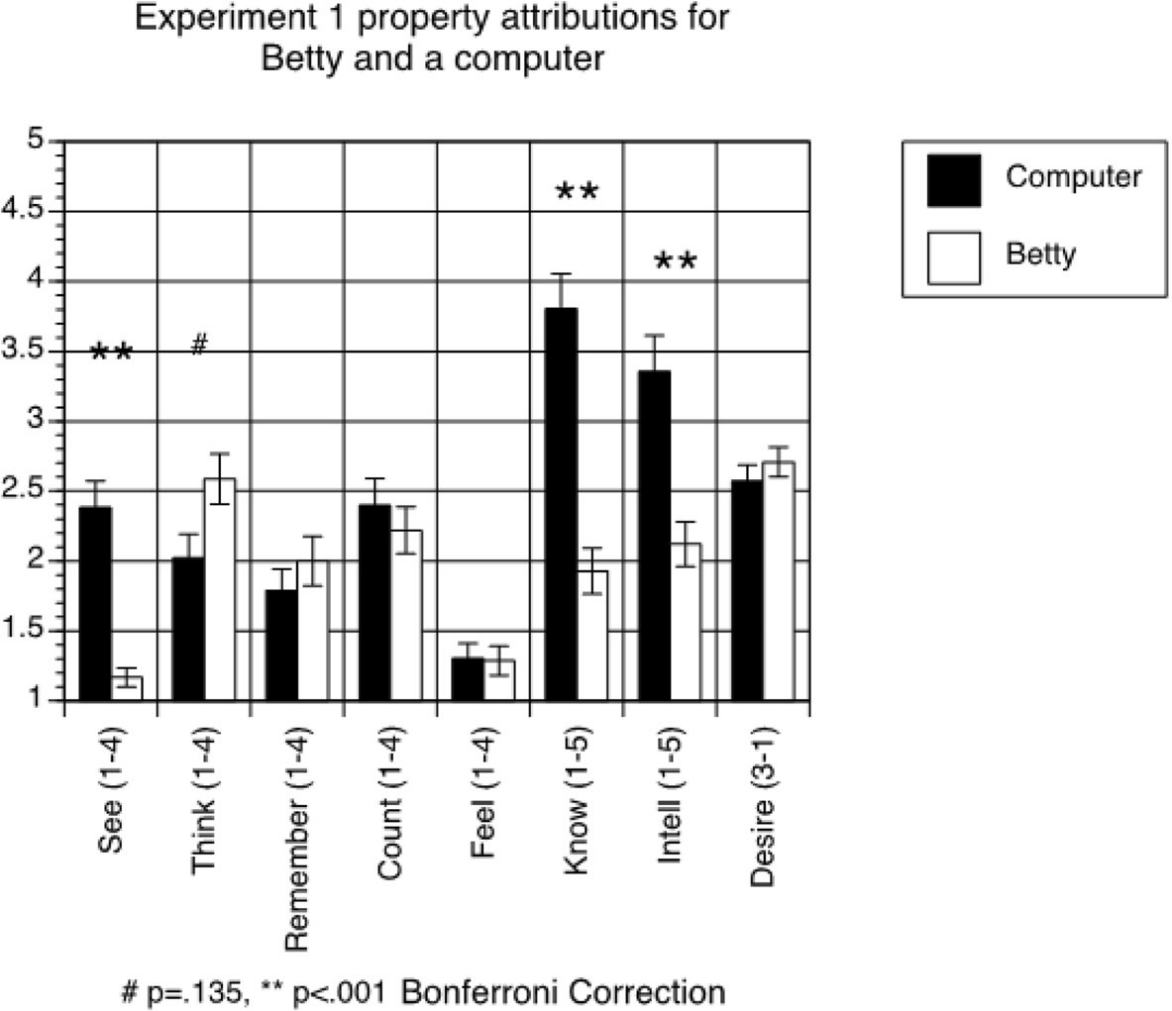
Results of property attribution questionnaire for Betty condition in Experiment 1. The error bars represent standard errors

Finally, we analyzed students’ responses to the eight-item property attribution questionnaire. We analyzed only responses from students in the experimental group (those who interacted with Betty), because only those students responded to the questionnaire for both Betty and a computer. A 2 × 8 within-subjects ANOVA (agent (Betty vs. computer) x question) revealed a significant main effect of agent (F(1,37) = 17.446, *p* < 0.001), with students generally attributing more human-like properties to a computer than to Betty (means = 2.455 and 1.994, respectively). The ANOVA also revealed a significant main effect of question (F(7,259) = 19.364, *p* < 0.001), and a significant interaction (F(7,259) = 16.828, *p* < 0.001). As shown Fig. 2, students believed the computer to be more knowledgeable, more intelligent, and more likely to see than Betty. In addition, students rated Betty as marginally more likely than the computer to think, though this difference fell short of statistical significance. There were no significant differences in students’ estimates of the computer’s and Betty’s abilities to remember, count, feel, and understand desire.

We also tested whether students’ property attributions might relate to or affect the observed link between behavioral predictions and learning. No individual items from the property attribution questionnaire predicted learning, so we calculated the mean of all of the eight ratings that were done for both the computer and Betty. This can be considered a measure of the degree to which participants globally attribute a range of intellectual skills to Betty and to a computer. We tested whether the mean property attributions were correlated with results of the behavioral predictions for humans, computers, and Betty, and found that they were not. In order to test for relationships between explicit attributions to software agents and content learning, we added mean property attributions for both Betty and the computer to the regression using person behavioral predictions to predict learning (i.e., students’ posttest scores, controlling for the corresponding pretest scores). The person behavioral predictions again significantly predicted learning (β = 0.268, *p* = 0.003), while students’ property attributions did not.

### Discussion

We found that students who made more intentional behavioral predictions for humans learned more effectively from the Betty’s Brain system. In addition, although the effect was not statistically significant in this experiment, students who used the Betty system made more intentional behavioral predictions for a human and fewer intentional behavioral predictions for a computer and for Betty than students who did not use the system. This could imply that using the Betty system refines children’s understanding of agency, helping them understand the overall differences between how human intelligence and machine intelligence manifest in specific situations. We revisit this topic in Experiment 2 below.

Finally, the pattern of results from the property attribution questionnaire allowed us to gain insight into how the students think about computers and Betty, and what their expectations of these agents might be. Interestingly, the students who had experience with Betty’s Brain rated Betty as being marginally more likely to think than the computer, while rating the computer as more intelligent and knowledgeable than Betty. Thus, it seems that the students who interacted with Betty began to consider her as separate from the hardware and programming, attributing a kind of independent information processing to her character. Betty’s lower intelligence and knowledge ratings may be due to her initial ignorance of the material (as the Betty agent only knows what she is taught by the student) and difficulties they may have experienced in trying to get Betty to give correct answers. In addition, students likely discovered that despite having an animated face, Betty could not see them, and therefore rated her ability to see as less than a computer which might be expected to at least process visual information via a web cam.

## Experiment 2

In order to examine the reliability of our Experiment 1 findings, a second experiment was conducted. The intervention was largely similar to that of Experiment 1, with some minor differences. First, some students used the version of Betty’s Brain system that was used in Experiment 1, while others used a new version. This new version was updated to provide more feedback to students. However, this updated version produced no detectable effects beyond the original Betty system and was therefore grouped together with the older version in all analyses.

Second, students in the control condition of Experiment 2 used a control version of the Betty’s Brain system that did not contain any agents. The use of this system allowed us to create a baseline condition that would isolate effects of using Betty’s Brain from the effects of using any educational computer software.

Third, the multiple choice component of the content pretest/posttest was modified from Experiment 1 to Experiment 2. Specifically, for Experiment 2, the multiple choice component was reduced from 14 items to 11 items, and was scored on an 11-point scale, with students receiving 1 point for each correct response and 0 points for each incorrect response.^4^

Fourth, in Experiment 2, all students underwent training in basic causal reasoning and completed causal reasoning pre- and posttests. In these tests, students answered questions about causal concept maps that were similar in form to those they created in Betty’s Brain, but concerning different content (for an example map and questions, see Additional file 1). We used these tests to examine whether any learning advantage associated with the behavioral prediction scenarios would be broad enough to include facilitation on causal reasoning tests. Several previous findings suggest this possibility. First, research exploring how knowledge supports abstract reasoning in adults suggests that many forms of basic causal reasoning are supported by relatively broad schemas (Cheng & Holyoak, 1985), and second, researchers exploring the development of causal reasoning in children have proposed a broad causal reasoning system that can take input from any of a number of more specific systems (Gopnik, Sobel, Schultz, & Glymour, 2001). On both of these views, it is possible that there will be a link between reasoning about how goals cause behavior on the behavioral prediction measure and a more general understanding of causal links.

### Method

#### Participants

We recruited 207 students from ten seventh and eighth grade classrooms in the same Nashville, Tennessee public middle school. A total of 136 students (97 experimental, 39 control) completed the study and were included in our analyses. Of the 136 students, 25% were classified as “honor students”, and 24 had previous experience with Betty’s Brain. The study underwent the same approval and consenting process as Experiment 1.

#### Materials

For this experiment, we included a causal reasoning training exercise and a causal reasoning pretest and posttest. The causal reasoning test was an 18-item multiple choice test in which students were asked to reason about causal maps ranging in complexity from two nodes and one link to ten-node maps in which distant nodes were connected by multiple links.

The content pretest and posttest were generally administered as they were in Experiment 1, with the modifications to the multiple choice component described above. The behavioral prediction questionnaire was administered just as it was in Experiment 1. The property attribution questionnaire was also the same as in Experiment 1 except that it contained nine items rather than eight. The additional item probed students’ attributions of semantic knowledge to an agent (a computer or Betty), asking whether the agent knows what things like sunshine and dogs are and what they are like.

Tennessee Comprehensive Achievement Program (TCAP) scores were collected for most participants. This is a standardized test issued to students in grades 3 through 8 in Tennessee. The TCAP is in multiple-choice format and covers reading, mathematics, science, and social studies.

#### Procedure

Students were assigned to one of three conditions by classroom. The control condition used a version of the Betty’s Brain software that contained no agents and issued no feedback^5^ but allowed the students to create concept maps. The first experimental condition used the same version of Betty’s Brain described in Experiment 1. The second experimental condition used an updated version of Betty’s Brain that provided more extensive feedback to the students but was otherwise very similar to the first experimental condition.^6^ Because the updated version of Betty’s Brain produced no effects that differentiated it from the original version, we group the two together as one experimental condition in all statistical analyses.

The classroom intervention schedule was identical to that of Experiment 1, except that all students received causal reasoning training before beginning work on their concept maps. During the training, students were walked through a 23-slide PowerPoint presentation that introduced concepts in causal reasoning using box-and-arrow diagrams. The presentation began with a basic description of nodes, as well as positive and negative links between nodes, and ended with examples of complex diagrams that included branching and multiple paths.

Students covered the same substantive material in all experimental conditions.

### Results

#### Behavioral predictions

As in Experiment 1, we conducted a 2 × 3 mixed ANOVA with condition (control vs. experimental) as a between-subjects factor, agent type (human vs. computer vs. Betty) as a within-subjects factor, and intentional behavioral predictions as the dependent variable. Similar to Experiment 1, we found no main effect of condition (F(1,134) = 0.096, *p* = 0.76) and a significant main effect of agent type (F(2,268) = 5.984, *p* = 0.003). With respect to agent type, students made more intentional behavioral predictions for the human agent (*M* = 62%, 1.87 of 3) than they did for Betty (*M* = 45%, 1.36 of 3; Bonferroni-corrected *p* = 0.009) or for the computer (*M* = 47%, 1.42 of 3; Bonferroni-corrected *p* = 0.018).

Importantly, our ANOVA also revealed a significant interaction between condition and agent (F(2, 268) = 5.225, *p* = 0.006). As predicted, students in the experimental condition drew significantly sharper distinctions between humans and machines than those who did not. Specifically, in the experimental condition, students made more intentional predictions for the person (*M* = 67%, 2.00 of 3) than for the computer (*M* = 47%, 1.40 of 3; Bonferroni-corrected *p* < 0.001) or for Betty (*M =* 42%, 1.27 of 3; Bonferroni-corrected *p* < 0.001), whereas in the control condition students’ intentional predictions for a person (*M =* 52%, 1.55 of 3) did not differ from their predictions for a computer (*M* = 49%, 1.46 of 3) or for Betty (*M* = 52%, 1.56 of 3). Table 1 above provides a summary of participants’ behavioral predictions split by condition and agent.

Because there may have been some baseline differences in test scores between the Betty and control conditions (Table 2), we verified that the effect of condition on intentional behavioral predictions remains even when controlling for TCAP scores, content pretest scores, and content posttest scores. We tested this using three follow-up regressions,^7^ each of which used one of these three control variables and condition to predict the difference between students’ intentional behavioral predictions for a person and their intentional behavioral predictions for machines (i.e., the average of intentional behavioral predictions for Betty and for a computer). All three models were significant (TCAP model, (F(2,113) = 3.865, *p* = 0.024; Content Pretest model, F(2,132) = 6.537, *p* = 0.002; Content Posttest model, F(2, 129) = 7.202, *p* = 0.001), and condition was a significant predictor in each (TCAP model, β = 0.213, *p* = 0.022; Content Pretest model, β = 0.223, *p* = 0.008; Content Posttest model, β = 0.223, *p* = 0.019). The same pattern results when the regressions are run with intentional behavioral predictions for a person as the outcome variable (TCAP model, F(2,113) = 4.561, *p* = 0.012; condition β = 0.237, *p* = 0.010; Content Pretest model, F(2,132) = 8.845, *p* < 0.001; condition β = 0.206, *p* = 0.013; Content Posttest model, F(2,129) = 7.091, *p* = 0.001; condition β = 0.197, *p* = 0.022).

#### Behavioral predictions and learning

Students’ performance on the content pre- and posttest is summarized in Table 2, and students’ performance on the causal reasoning pre- and posttest is summarized in Table 4. With respect to the content test, paired t tests revealed that students in both conditions improved significantly from pretest to posttest on the multiple choice component (Betty: t(91) = 5.06, p < .001; control: t(37) = 3.93, p < .001), but not on the short answer component (Betty: t(82) = 0.97, *p* = .33; control: t(33) = 2.28, *p* = .03). With respect to causal reasoning test, students in both conditions improved significantly from pretest to posttest (Betty condition: t(89) = 3.09, *p* = .003; control condition: t(33) = 2.28, p = .03).

**Table 4:** Mean scores on causal reasoning (*CR*) pretests and posttests for Experiments 2–3

	CR Pre	CR Post
Experiment 2		
Control (*N* = 39)	6.28 (3.06)	7.57 (3.63)
Betty (*N* = 97)	8.29 (3.73)	9.22 (3.82)
Experiment 3		
Betty (*N* = 75)	1.83 (1.25)	2.90 (1.06)

Standard deviations appear in parentheses after means. Note that Experiment 2 features an 18-item causal reasoning pre- and posttest, while Experiment 3 featured a condensed six-item version

In this experiment, there were between-condition differences on the content posttests. Specifically, students in the Betty condition scored higher on the content posttest than students in the control condition, t(130) = 1.996, *p* = .048, and did not score significantly higher on the content pretest, t(97.90) = 1.141, *p* = .257. However, improvement from content pretest to content posttest was generally similar across conditions (t(100.46) = 1.185, *p* = .239). Students in the Betty condition also scored higher than students in the control condition on both the causal reasoning posttest, t(126) = 2.25, *p* = .026, and the causal reasoning pretest, t(76.16) = 3.167, *p* = .002. Again, improvement from pretest to posttest was similar across conditions, t(122) = −.705, *p* = .482.

As with Experiment 1, we tested for relationships between intentional behavioral predictions and learning outcomes. We ran three regressions that paralleled those we ran in Experiment 1, with each regression including behavioral predictions for one of the three agents (human, Betty, or computer), condition, and content pretest scores and as predictors of content posttest scores. We also ran a second set of three regressions that included behavioral predictions, condition, and *causal reasoning* pretest scores as predictors of *causal reasoning* posttest scores.

Intentional behavioral predictions did not significantly predict content posttest scores in this experiment.^8^ However, as reported in Table 5, intentional behavioral predictions for a person were a significant predictor of causal reasoning posttest scores, controlling for causal reasoning pretest scores. To test whether the relationship between person behavioral predictions and learning varied across conditions, we re-ran our regression with a prediction-condition interaction term added to the model. The interaction was not statistically significant (β = .150, *p* = .27). However, similar to Experiment 1, intentional behavioral predictions for a person significantly predicted causal reasoning posttest scores in the experimental condition and not in the control condition (Table 5).

**Table 5:** Regressions predicting causal reasoning posttest scores using causal reasoning pretest scores, condition, and intentional behavioral predictions for a person in Experiment 2

	Beta	*t*	*p*
Person behavioral predictions			
Overall R^2^ = 0.474, F(3,120) = 35.976, *p* < 0.001			
Person behav. pred.	0.175	2.492	0.014
Pretest	0.628	8.975	< 0.001
Condition	− 0.016	− 0.228	0.820
Betty condition only R^2^ = 0.542, F(2,87) = 51.406, *p* < 0.001			
Person behav. pred.	0.213	2.889	0.005
Pretest	0.666	9.014	< 0.001
Control condition only R^2^ = 0.225, F(2,31) = 4.502, *p* = 0.019			
Person behav. pred.	0.050	0.308	0.760
Pretest	0.462	2.863	0.007

The “Betty Condition Only” and “Control Condition Only” follow-up regressions include only participants in the named condition

One possible explanation of our findings to this point is that students with higher general intelligence both learn more about causal reasoning from the Betty’s Brain system and make better behavioral predictions for a person. To address this possibility, we conducted a new regression, predicting students’ causal reasoning posttest scores in the experimental (Betty) condition, controlling for students’ TCAP scores in reading, math, science, and social studies, in addition to causal reasoning pretest scores. Intentional behavioral predictions for a person again significantly predicted learning in this regression (β = 0.235, *p* = 0.004), while none of the TCAP scores were significant predictors.^9^ This provides evidence against the idea that the relationship between person behavioral predictions and learning is simply mediated by general intelligence.

Finally, we ran two additional regressions on our full sample to examine whether person behavioral predictions were predictive of content posttest scores for either the multiple choice or short answer component, controlling for the corresponding pretest score. Person behavioral predictions were significant predictors for the short-answer questions (β = 0.205, *p* = 0.016), but not the multiple-choice questions (β = 0.074, *p* = 0.354).

#### Property attribution questionnaire

**Fig. 3:**
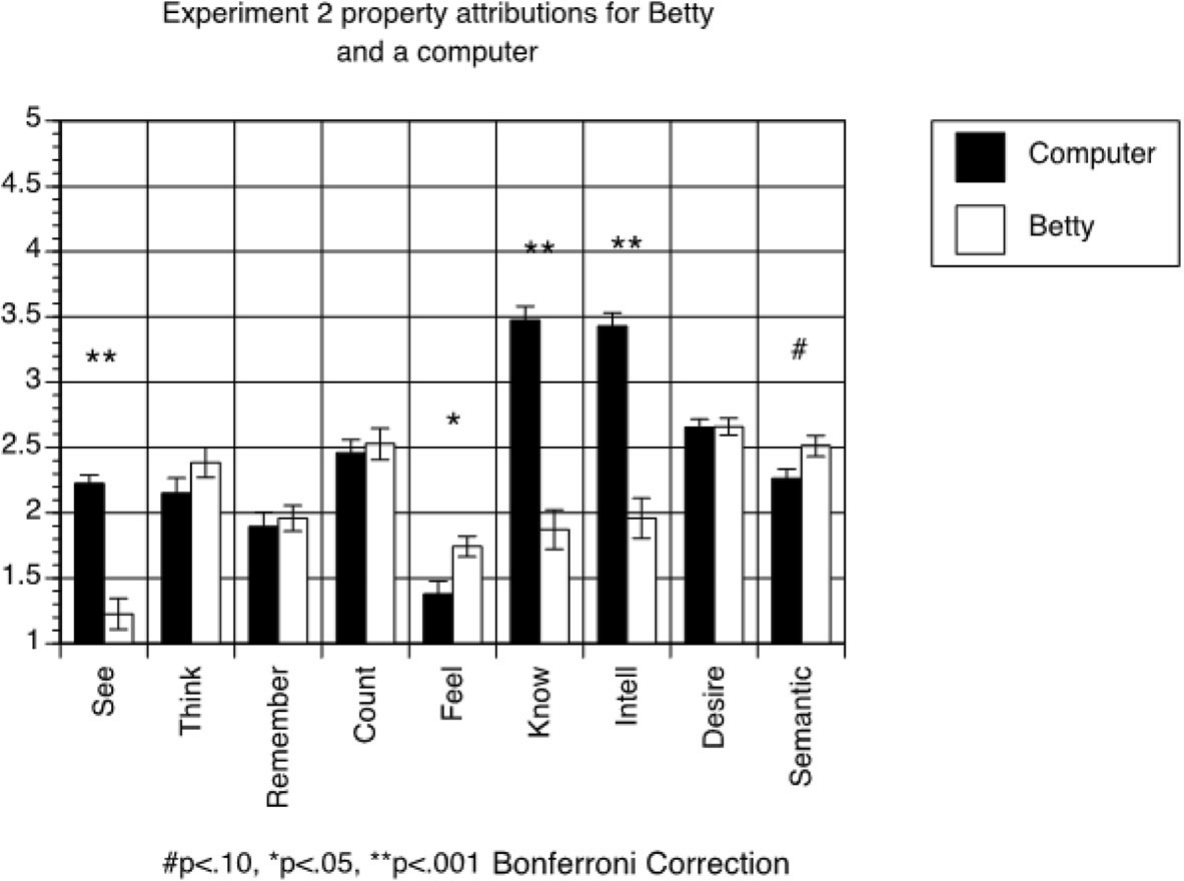
Results of property attribution questionnaire for Betty condition in Experiment 2. The error bars represent standard errors

A 2 × 9 within-subjects ANOVA (agent (Betty vs. computer) x question) revealed a significant main effect of agent (F(1,91) = 36.151, *p* < 0.001), with students generally attributing more human-like properties to a computer than to Betty (means = 2.448 and 2.043, respectively). Further, the ANOVA revealed a significant main effect of question (F(7,637) = 37.031, *p* < 0.001), and a significant interaction (F(7,637) = 41.122, *p* < 0.001). As shown in Fig. 3, students believed the computer to be more knowledgeable, more intelligent, and more likely to be able to see than Betty. Students also reported that Betty was more likely to have feelings than a computer.

In addition, as in Experiment 1, we calculated the means of all of the property attribution ratings for both the computer and Betty and found those means did not correlate with behavioral predictions for any of the agents. The relationship between person behavioral predictions and learning on the causal reasoning measure remained significant when controlling for average property attributions to Betty (β for person behavioral predictions = 0.213, *p* = 0.005; β for property attribution ratings for Betty = 0.010, *p* = 0.892). The same was true when controlling for average property attributions to the computer (β for person behavioral predictions = 0.216, *p* = 0.004; β for property attribution ratings for the computer = 0.083, *p* = 0.258).

#### Actions associated with success using the Betty system

Experiments 1 and 2 both provide evidence that pragmatic understanding of human agency predicts successful use of an agent-based tutoring system. This led us to ask whether students with stronger pragmatic understandings of human agency used the Betty’s Brain software differently than other students. A median split was used to divide the Experiment 2 participants into a “High Intentionality” group (students who believed the human agent to have more intentional representation) and a “Low Intentionality” group (students who believed the human agent to have less intentional representation) based on their behavioral predictions. Students in the High Intentionality group more frequently queried the software agent (high intentionality mean = 21.37 and low intentionality mean = 9.07, t(41.53) = 2.84, *p* = 0.007). Queries include voluntary interactions with the agent, such as asking Betty a question about the relationship between concepts, which she then answers using the student’s concept map. The difference was specific to queries: the High Intentionality group and Low Intentionality group did not differ with respect to the overall number of actions taken in the Betty’s Brain system (total actions include not only queries but reading passages, editing causal maps, etc.).

Human behavioral predictions remained a significant predictor of causal reasoning learning in the Betty condition when controlling for number of queries (β = 0.217, *p* = 0.006), and the number of queries was not a significant predictor in this model, β = − 0.018, *p* = 0.86. Similarly, human behavioral predictions remained a significant predictor of learning when controlling for the overall number of actions students took in the Betty’s Brain system (β = 0.221, *p* = 0.005), and the total number of actions taken was not predictive, β = 0.095, *p* = 0.21. Thus, it is unlikely that the relationship between intentional behavioral predictions for humans and increased learning from Betty’s Brain is simply a consequence of engagement or number of interactions with the agent.

### Discussion

As in Experiment 1, students who made more intentional behavioral predictions for a person tended to learn more from using the Betty’s Brain system. In Experiment 2, however, this effect manifested in the causal reasoning test (where it was present even when controlling for TCAP scores, when controlling for the number of times students queried Betty, and when controlling for the number of actions that students made in the Betty’s Brain system). The relationship between behavioral predictions and learning on the content test did not replicate in Experiment 2, although intentional predictions for a person were significant predictors of learning on the short-answer component. We defer discussing this in depth until the results of Experiment 3 have been presented to reinforce the consistency of the link between behavioral predictions and posttest performance.

Additionally, in Experiment 2, students who interacted with Betty demonstrated stronger pragmatic understandings of agency than students who did not. That is, students in the Betty condition distinguished between human behavior and machine behavior on the behavioral prediction questionnaire *more* than their counterparts in the control condition. While this pattern was present but not statistically significant in Experiment 1, it was statistically significant in Experiment 2 (*p* = 0.004). The difference was driven in part by a smaller proportion of intentional predictions for Betty in the experimental condition, but also in part by a higher proportion of intentional predictions for the person in the experimental condition. This is consistent with the idea that experience with ambiguous agents like Betty brings the practical distinction between intentional and mechanical conduct into sharper relief. It could also be the case that the smaller proportion of intentional predictions for Betty in the experimental group is related to students’ impressions of Betty’s intelligence and knowledge (which they rated significantly lower than the computer in both Experiments 1 and 2).

Finally, with respect to the property attribution questionnaire, students in Experiment 2 again signified beliefs about Betty’s agency that go beyond her software implementation. Students again rated a computer as more intelligent and knowledgeable than Betty. Further, students rated Betty as more likely to “feel” than a computer.

## Experiment 3

Experiment 3 had two basic purposes. First, because of inconsistency in the specific learning outcomes associated with intentional behavioral predictions, we wanted to test again whether scores on content and causal reasoning tests were related to behavioral predictions. Second, in both Experiments 1 and 2, the behavioral prediction questionnaire was given *after* the students used the Betty system. Therefore, it is possible that individual differences in performance on the Betty exercise led to differences in intentional predictions on the behavioral prediction questionnaire. To address this possibility, participants in Experiment 3 completed the behavioral prediction scenarios *before* completing the Betty exercise. Experiment 3 was otherwise similar to Experiment 2, except that the causal reasoning pretest/posttest was condensed from 18 items to 6 and all students in Experiment 3 used the Betty system with agents.

### Method

#### Participants

A total of 75 fifth and sixth grade students from the same Nashville, Tennessee middle school completed the experiment. Of these, 71 completed the Betty’s Brain content pre- and posttests. The study underwent the same approval and consenting process as Experiments 1 and 2.

#### Materials

Materials used were the same as in Experiment 2.

#### Procedure

Procedures for Experiment 3 were similar to Experiment 2 with three exceptions. First, students used only the Betty system with agents. There were no controls. Second, the causal reasoning pretest/posttest was condensed from 18 items to 6. Third, and most importantly, students completed the behavioral prediction scenarios before, rather than after, the teachable agent learning sessions.

### Results

#### Behavioral predictions

A one-way repeated-measures ANOVA revealed that participants’ behavioral predictions varied across agent types (F(1,74) = 581.80, *p* < 0.001). Students made, on average, 72% (2.17 of 3) intentional behavioral predictions for a person, 43% (1.29 of 3) for a computer, and 50% (1.51 of 3) for Betty. Post hoc tests revealed that participants made significantly more intentional predictions for the person than for the computer (Bonferroni-corrected *p* < 0.001) or for Betty (Bonferroni-corrected *p* < 0.001).

As noted above, all participants in Experiment 3 used a version of the Betty system that included a teachable agent. As a result, we could not test the effect of using the Betty system on students’ behavioral predictions in this experiment.

#### Behavioral predictions and learning

Students’ performance on the content pre- and posttest is summarized in Table 2, and students’ performance on the causal reasoning pre- and posttest is summarized in Table 4. With respect to the content test, paired *t* tests revealed that students improved from pretest to posttest on both multiple choice questions (t(70) = 6.28, *p* < 0.001) and short answer questions (t(70) = 4.09, *p* < 0.001). With respect to the causal reasoning test, another paired *t* test revealed significant improvement (t(70) = 5.92, *p* < 0.001).

As shown in Table 6, person behavioral predictions again predicted learning on the causal reasoning test. Person behavioral predictions were also nearly significant predictors of learning on the multiple choice component of the content test, but not the short answer component. In no case did behavioral predictions for Betty or the computer predict learning.

**Table 6:** Regressions predicting causal reasoning and content posttest scores using the corresponding pretest scores and intentional behavioral predictions for a person in Experiment 3

	Beta	*t*	*p*
Person behavioral predictions			
Causal reasoning posttest R^2^ = 0.089, F(2,68) = 3.340, *p* = 0.041			
Person behav. pred.	0.265	2.293	0.025
Pretest	0.138	1.193	0.237
Content multiple choice posttest R^2^ = 0.445, F(2,68) = 27.237, *p* < 0.001			
Person behav. pred.	0.169	1.833	0.071
Pretest	0.614	6.669	< 0.001
Content short answer posttest R^2^ = 0.101, F(2,68) = 3.800, *p* = 0.027			
Person behav. pred.	0.076	0.658	0.512
Pretest	0.297	2.558	0.013

#### Property attribution questionnaire

**Fig. 4:**
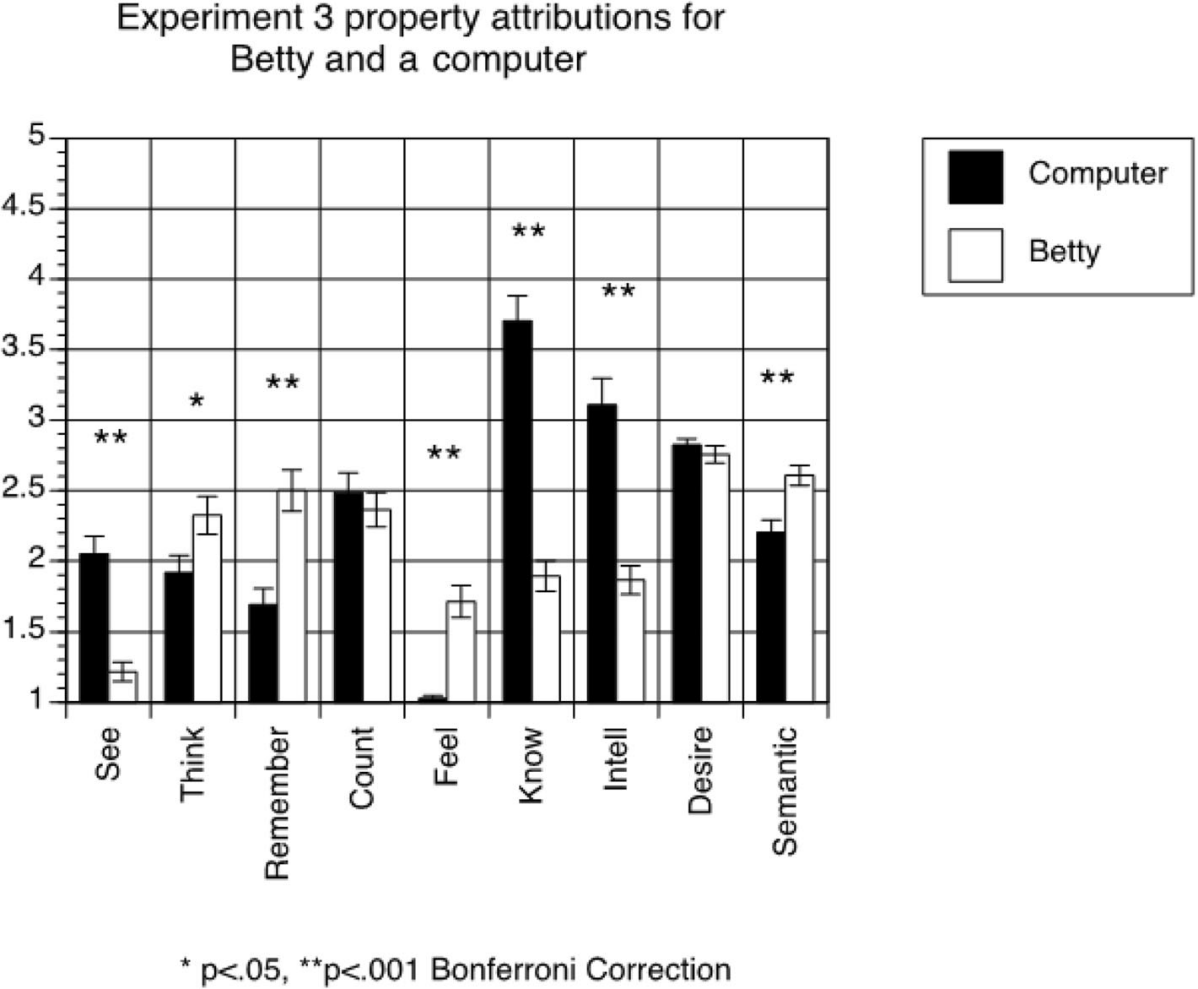
Results of property attribution questionnaire in Experiment 3. The error bars represent standard errors

A 2 × 9 within-subjects ANOVA (agent (Betty vs. computer) x question) revealed a significant main effect of agent (F(1,72) = 10.148, *p* = 0.002), with students generally attributing more human-like properties to a computer than to Betty (means = 2.335 and 2.139, respectively). The ANOVA also revealed a significant main effect of question (F(8,576) = 31.855, *p* < 0.001), and a significant interaction between agent and question (F(8,576) = 43.900, *p* < 0.001). As shown in Fig. 4, students rated the computer as more likely to see, more intelligent, and more knowledgeable than Betty. In contrast, students believed that Betty was more likely to think, to remember, to have feelings, and to have semantic representations than a computer.

Similar to the previous studies, we calculated the mean of all property attribution ratings for Betty and for the computer and tested for relationships between these means and behavioral predictions. We found none. The relationship between person behavioral predictions and learning causal reasoning remained significant when controlling for average property attributions to Betty (β for person behavioral predictions = 0.272, *p* = 0.023; β for property attribution ratings for Betty = 0.060, *p* = 0.612). The same was true when controlling for average property attributions to the computer (β for person behavioral predictions = 0.268, *p* = 0.026; β for property attribution ratings for the computer = − 0.023, *p* = 0.846).

### Discussion

Experiment 3 largely replicated Experiment 2. Students who made more intentional behavioral predictions for a person again tended to learn more through use of the Betty’s Brain system, and those effects again manifested on the causal reasoning test. Person behavioral predictions were again marginally predictive of learning on one component of the content test, though in this experiment it was the multiple choice component. For the third time in three studies, there was no relationship between participants’ explicit property attributions and posttest performance, nor between their explicit property attributions and their behavioral predictions. Finally, participants continued to distinguish between Betty and a computer in the property attribution questionnaire, and continued to rate Betty as less intelligent, less knowledgeable, and less likely to see than a computer.

## General discussion

The three experiments reported here produced several basic findings about the connections between students’ use of agency concepts and learning from teachable software agents. From our perspective, the most important finding is the consistent link between students’ learning outcomes with the Betty’s Brain system and their pragmatic understanding of *other people’s* agency (i.e., the number of intentional behavioral predictions they made about a human agent). All three experiments demonstrated a link between behavioral predictions for a human and one or more measures of learning. But it is not clear why this link was strongest for learning about causal reasoning (observed in Experiments 2 and 3) and less consistent for learning about science content.^10^ This may simply be due to variation that can be expected across any set of attempts to observe moderate relationships near the limits of statistical detectability. In addition, it appears as though the overall amount of learning was sometimes modest, making it difficult to observe strong relationships between learning and any other measure. So, we would argue that the link between an understanding of human agency and content learning from teachable-agent-based software should be considered tentative, but, in our view, likely, given the pattern of findings across these experiments (see Table 7 for a summary of significant findings across experiments).

**Table 7:** Summary of findings across experiments

	Experiment 1	Experiment 2	Experiment 3
Behavioral predictions			
Differ across agent?	***	**	***
Differ across conditions?	*ns*	*ns*	N/A
Agent x condition interaction?	*ns*	**	N/A
Person behavioral predictions and learning			
Predict content learning?			
Multiple choice	**	*ns*	#
Short answer	*	*	*ns*
Overall	**	*ns*	*ns*
Predict causal reasoning learning?	N/A	*	*
Property attribution questionnaire			
Differ across agent?	***	***	**
Differ across properties?	***	***	***
Agent x property interaction?	***	***	***
	Students rated computer as more likely to: See Be intelligent Know things	Students rated computer as more likely to: See Be intelligent Know things	Students rated computer as more likely to: See Be intelligent Know things
		Students rated Betty as more likely to: Feel Have semantic knowledge	Students rated Betty as more likely to: Feel Have semantic knowledge Remember Think

#*p* = 0.071, *p < 0.05, **p < 0.01, ***p < 0.001; *ns* not significant

One question about this link is *why*? That is, why would behavioral predictions about a person relate most closely to learning from the Betty system when predictions about Betty or a computer might seem to be more likely candidates given their similarity with the features of the learning system?

Students who can understand and predict the actions of intentional agents could be at an advantage in learning because they are better able to understand how Betty operates. After all, Betty is, in many respects, programmed to act like a human. Thus, students who more readily deploy concepts about intentional, “human-like” behavior in novel situations may be quicker to understand Betty’s behavior (e.g., why Betty misses a question in a quiz) and, more broadly, how the Betty’s Brain system is intended to work. In a sense, these students may be better at “playing along” with the idea that Betty is human in the context of the educational software, strategically applying intentional agency concepts to facilitate learning.

Alternatively, it may be that deploying the appropriate concepts of agency at the correct time and monitoring cues from the Betty software to determine which concepts are most helpful in what circumstances taxes students’ cognitive resources. Perhaps students who more readily understand the behaviors of intentional agents can apply the appropriate concepts to Betty using fewer cognitive resources. This would leave more resources available for the metacognitive monitoring at the heart of the learning-by-teaching paradigm—monitoring that Betty is specifically designed to elicit and that is known to be resource-intensive (Schwartz et al., 2009). In short, then, perhaps students with stronger pragmatic understandings of agency can spend fewer resources on understanding Betty and more resources on learning class material. This would be consistent with research from Herberg et al. (2012), who found that explaining the solution to a problem to a nominally intentional audience can sometimes interfere with subsequent performance.

Regardless of the precise mechanism, our results indicate that a pragmatic understanding of agency facilitates learning from software agents. This pragmatic understanding extends beyond basic concepts of goal-directed behavior, as evidenced by the variability in students’ responses on the behavioral prediction questionnaire. We suggest it is useful to think of this variability as reflecting differences in “agent fluency”. Knowing basic object labels in a second language does not necessarily mean that one can converse fluently with a native speaker. Similarly, while middle school students undoubtedly grasp basic concepts of goal-directedness and intentionality, they vary in their elaboration of these concepts and their ability to effectively apply them to make predictions in specific novel situations (as do adults; Levin, Saylor, et al., 2013).

It is interesting that students’ behavioral predictions were most consistently predictive of scores on the causal reasoning test. Prior research has shown that students tend to learn causal reasoning from Betty’s Brain (Chin et al., 2010), but to our knowledge no prior research has linked causal learning from a software agent to learners’ concepts of agency. One possible explanation of this link is that the broad reasoning skills underlying theory of mind development can encompass both reasoning about agents and more abstract causal reasoning (Gopnik et al., 2001). On this account, skills useful in understanding the intentionality of a novel, somewhat-anthropomorphic agent like Betty also facilitate learning the novel causal reasoning scheme instantiated in the causal reasoning pre- and posttests. However, future research could contrast this hypothesis with a more general resource allocation hypothesis.

In addition to demonstrating links between pragmatic understanding of agency and learning, we have also collected data demonstrating a relationship between interaction with a software agent and pragmatic understanding of agency. Specifically, we found evidence that experience with the Betty system increased the contrast between students’ behavioral predictions for people and their predictions for machines. In Experiment 1 this difference was not statistically significant, but in Experiment 2 there was a significant increase in human–machine differentiation in students who used the Betty system relative to a no-Betty control.

Thus, there appears to be a two-way relationship between concepts about agents and learning. This is particularly interesting in view of the hypothesis that interaction with novel software agents, such as robots, may induce a broad revolution in understanding of agency, and, more generally, life itself (Kahn, Friedman, Perez-Granados, & Freier, 2006). Although this kind of broad revolution is possible, such revolutions are not often observed, and a more modest hypothesis is that experience with software agents will help learners clarify their existing understanding of what it means to be human and to think, or perhaps facilitate shifts among different existing understandings (e.g., Taber, 2001). One reason to be skeptical of the possibility of a broad conceptual revolution is that, in our studies, students’ behavioral predictions were uncorrelated with explicit attributions to the agents on the property attribution questionnaire. Therefore, it may be that the conceptual change observed here avoids contact with explicit beliefs.

One potential limitation of our studies is that our measure of pragmatic understanding of agency is not narrowly tailored to the particular capabilities of the agent Betty (or of teachable agents in educational software more generally). While probing students’ predictions about Betty’s ability to reason causally or use strategies to meet goals would be quite interesting, our use of a broader measure was deliberate. We chose a broader measure because we were interested in how even very broad agency concepts are called upon for, and affected by, specific interactions with particular agents, and because our findings using a broader measure are more likely generalizable to other contexts involving technological agents.

Another important limitation inherent to the design of our study is that it is difficult to conclusively demonstrate that the agent concepts tapped by the behavioral prediction questionnaire *caused* improved learning. Given that we only assessed individual differences in behavioral predictions and did not actually manipulate them, it is possible that an unmeasured third factor caused both intentional predictions for people and success in learning from the Betty system. In Experiment 2 we were able to control for broad academic achievement scores, so these seem like an unlikely third factor. However, theory of mind is often linked with social and language skills (for example, Garfield, Peterson, & Perry, 2001), and these may not be well-represented in academic achievement tests. Thus, it is possible that these broad skills underlie both intentional behavioral predictions and success with the Betty system. Although we would consider this a psychologically interesting finding, we would nonetheless also advocate for follow-up research that could more directly test causal hypotheses. It would be interesting to assess whether training students in the pragmatic engagement of agency concepts would lead to improved learning from the Betty system. This would not only be theoretically interesting, but it could lay the basis for a successful intervention aimed at improving student success with agent-based learning aids.

## Conclusion

These three experiments represent the first assessment of links between agency concepts and learning from a computer-based teachable-agent learning system. All three experiments revealed that students with a stronger pragmatic understanding of human agency experienced greater learning outcomes after using the Betty’s Brain teachable-agent system. Combined with findings that use of the system in turn refines students’ understanding of agency, our results represent an important first step toward understanding how knowledge about agents is leveraged to support learning in technological contexts.

More broadly, the use of teachable agent systems like Betty’s Brain is only one of an increasing variety of contexts in which people interact with software agents. This paper provides an initial description of how agency concepts shape, and are shaped by, these interactions. As software agents become engrained in our daily lives, we can benefit from a detailed, explicit understanding of how we interact with them—and how those interactions might affect us.

## Additional file

Additional file 1: Sample behavioral prediction scenarios and sample content questions. (DOCX 4003 kb)

## Footnotes

1 For a more specific description of how the Betty’s Brain system works, see the “Materials” section of Experiment 1.

2 Specifically, to quantify learning, we examine variability in students’ posttest scores controlling for the corresponding pretest scores.

3 Prior to administering the content pretest, the multiple choice questions were classified as one of three types: identification questions (four questions, worth 1 point each), definition questions (five questions, worth 2 points each), and explanation questions (five questions, worth 4 points each). This scoring system was established in accordance with the goals of another research project using the same data set.

4 This change in scoring system was motivated by the goals of another project using the same data set and was made prior to collection and analysis of data.

5 An experimenter provided all feedback in the control condition.

6 Specifically, the feedback provided to students in the enhanced version was altered to provide more specific suggestions as to how students should proceed. Further, students in the enhanced version were provided with additional feedback if they were not showing improvement over time or appeared to be making uninformed edits to their concept maps. Finally, the enhanced version afforded students more interactions with Betty in two respects: (i) students were allowed to evaluate Betty’s answers and (ii) Betty queried students’ confidence after students made edits to their concept maps.

7 We ran three separate regressions to avoid multicollinearity problems, as TCAP scores, content pretest scores, and content posttest scores were correlated with one another (all *r*’s > 0.578).

8 Intentional behavioral predictions for the person came closest to significance, β = 0.096, *p* = 0.15.

9 A regression run on the full sample (both the experimental and control conditions) reveals the same pattern of results, with intentional behavioral predictions for a person significantly predicting learning (β = 0.194, *p* = 0.009). However, looking only at students in the control condition, intentional behavioral predictions for a person were not a significant predictor of learning (β = 0.046, *p* = 0.81).

10 Person behavioral predictions were predictive of overall content learning in Experiment 1, but were only predictive of short answer improvement in Experiment 2 and nearly predictive of multiple choice improvement in Experiment 3.
