# Supplementary material for: The interrelationship between concepts about agency and students’ use of teachable-agent learning technology
Source: Cogn Res Princ Implic. 2019 Apr 18;4:14. doi: 10.1186/s41235-019-0163-6 (PMC6473007; doi:10.1186/s41235-019-0163-6)
Supplement: Supplementary file 1 — Sample behavioral prediction scenarios and sample content questions. (DOCX 4003 kb) [file 41235_2019_163_MOESM1_ESM.docx]

Additional file1

Sample Content Questions

Multiple Choice Questions

1. What is the greenhouse effect?
   1. The atmosphere of the earth traps some heat energy and prevents it from being released into space. This makes the earth warmer.
   2. The atmosphere of the earth is reflective like the glass of a greenhouse. The light reflection keeps the earth from getting too hot.
   3. The atmosphere acts like a magnifying glass. This makes the light stronger and makes the earth hotter.
   4. The atmosphere traps pollution from cars and factories. Over time, the air will become more polluted and the earth will get warmer.
2. Which of these gases is a greenhouse gas?
   1. Nitrogen
   2. Carbon dioxide
   3. Oxygen
   4. All of the above

Short Answer Questions

1. We now know that deforestation, i.e., cutting of a large number of trees increases global temperature. Can you clearly list step-by-step the chain of events that explains how deforestation increases global temperature.
2. Scientists and engineers have invented exciting new ways to make more affordable powerful batteries! This makes it possible for more people to use electric cars, instead of regular cars that use gasoline and produce carbon dioxide. Please explain, step-by-step, how this invention could influence climate change.

Sample Causal Reasoning Questions

How does your body help you stay warm when it is cold outside? The way your body stays warm is a process called “thermoregulation.” This diagram shows some of the ways your body helps keep your body temperature up on cold days. Use this diagram to answer the questions below.


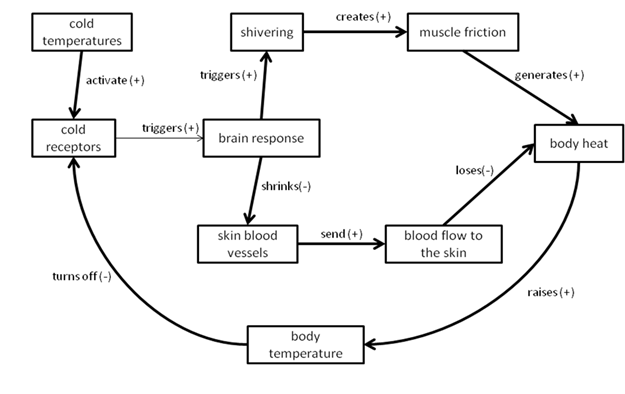


1. If shivering decreased, what would happen to muscle friction?
2. Muscle friction would increase.
3. Muscle friction would decrease.
4. Muscle friction would stay about the same.

Using the map, give a step-by-step explanation for how decreased shivering would cause this happen.

1. If blood flow to the skin increased, what would happen to body temperature?
2. Body temperature would increase.
3. Body temperature would decrease.
4. Body temperature would stay about the same.

Using the map, give a step-by-step explanation for how increased cold receptors would cause this happen.

Sample Behavioral Prediction Questions

| For the next exercise, think about what a person would do. Think of how a person acts and what makes them work. | **** |
| --- | --- |
| A duck and a truck are shown to a person. They are placed like this on a grid.  First, the person chooses the duck at A-1. | ****** |
| Next, the person chooses the duck at A-1 again. | ****** |
| Now, the duck and truck are switched.  Will ­­­­­­­­­­­­­­­­­­­­the person choose the truck at A-1, or the duck at C-3.  Circle your answer.  1. The person will choose the truck at A-1  2. The person will choose the duck at C-3. | **** |

| For the next exercise, think about what a computer would do. Think of how a computer acts and what makes them work. | **** |
| --- | --- |
| These items are shown to a computer.  They include a pen, a spoon, another pen, scissors, a pencil, a marker, and a screwdriver.  First, the computer chooses the first item, a pen. | **** |
| Next, the computer chooses the third item, another pen. | **** |
| Then, the computer chooses the fifth item, a pencil. | **** |
| Now, will the computer choose the sixth item, which is the marker, or the seventh item, which is the screwdriver?  Circle your answer.  1. The computer will choose the marker.  2. The computer will choose the screwdriver. | **** |

| For the next exercise, think about what Betty would do. Think of how Betty acts and what makes her work. | **** |
| --- | --- |
| These items are shown to Betty.  They include a tape dispenser, a bite-sized candy bar, a stapler, a roll of hard candy, gum, and a glue stick. | **** |
| If Betty were to put the items into two groups, would Betty organize the items like picture number 1, or picture number 2?  Number 1 Number 2 | |
| **** | **** |
